# Type 1 diabetes care delivery in Yaoundé, Cameroon: Social and political representations

**DOI:** 10.4102/phcfm.v16i1.4229

**Published:** 2024-03-15

**Authors:** Hervé B. Djiofack Kentsop, Christina Zarowsky, Julia E. von Oettingen

**Affiliations:** 1 Department of Social and Preventive Medicine, Faculty of Public Health, University of Montreal, Montreal, Canada; 2 Public Health Research Center, Faculty of Public Health, University of Montreal, Montreal, Canada; 3 CIUSSS du Centre-Sud-de-l’Île-de-Montréal, Montreal, Canada; 4 Research Institute, Health Centre, McGill University, Montreal, Canada; 5 Department of Pediatrics, Division of Pediatric Endocrinology, McGill University Health Centre, Montreal, Canada

**Keywords:** type 1 diabetes, chronic disease, Cameroon, health care system, care delivery, patient-centred care

## Abstract

**Background:**

Increasing chronic diseases challenges the health systems of low- and middle-income countries, including Cameroon. Type 1 diabetes (T1D), among the most common chronic diseases in children, poses particular care delivery challenges.

**Aim:**

We examined social representations of patients’ roles and implementation of T1D care among political decision-makers, healthcare providers and patients within families.

**Setting:**

The study was conducted in Yaoundé, Cameroon.

**Methods:**

Eighty-two individuals were included in the study. The authors conducted semi-structured interviews with policy makers (*n* = 5), healthcare professionals (*n* = 7) and patients ’parents (*n* = 20). Questionnaires were administered to paediatric patients with T1D (*n* = 50). The authors also observed care delivery at a referral hospital and at a T1D-focused non-governmental organisation over 15 days. Data were analysed using thematic content analysis and descriptive statistics.

**Results:**

Cameroonian health policy portrays patients with T1D as passive recipients of care. While many practitioners recognised the complex social and economic determinants of adherence to T1D care, in practice interactions focused on specific biomedical issues and offered brief guidance. Cultural barriers and policy implementation challenges prevent patients and their families from being fully active participants in care. Parents and children prefer an ongoing relationship with a single clinician and interactions with other patients and families.

**Conclusion:**

Patients and families mobilise experience and lay knowledge to complement biomedical knowledge, but top-down policy and clinical practice limit their active engagement in T1D care.

**Contribution:**

Children with T1D and their families, policy makers, healthcare professionals, and civil society have new opportunities to contribute to person-centred care, as advocated by the Sustainable Development Goals.

## Introduction

The rise in chronic diseases challenges the health systems in low- and middle-income countries. In sub-Saharan Africa, including Cameroon, health systems are largely organised around acute and infectious diseases and childbirth, and are consequently little prepared for involving patients in long-term follow-up.^1^ Recognising the experiential knowledge and patients’ self-management capacities implies a redefinition of the role of patients in medical work.^2^ The World Health Organization (WHO) called for a paradigm shift towards patient-centred care in 2003.^3^ Four groups of actors are key to this change: political decision-makers, who must adopt favourable strategies; health care providers, as promoters of care involving patients; the communities involved in prevention and support activities for affected patients; and the patients themselves, who must play an active role in their care. This call for a paradigm shift concerns all chronic conditions including so-called severe non-communicable diseases (NCDs) such as type 1 diabetes (T1D).^3^

Childhood chronic disease creates unique challenges and stressors for patients and families.^4^ Type 1 diabetes is among the most common childhood chronic diseases,^4^ with a substantial risk of mortality and morbidity. Successful treatment aims to reduce the risk of acute and chronic complications while preserving quality of life. To achieve this, intensive, ongoing patient and family education, daily disease self-monitoring and care interventions, and frequent medical follow-up are required. Further, policy makers must have sufficient awareness and health professionals must be adequately trained to be able to provide the support needed for intensive, complex and long-term care. In Cameroon, the T1D situation until 2010 was most likely similar to that described by Sap et al.^5^ in 1992 for sub-Saharan Africa. The reported prevalence of T1D was low, presumably due at least in part to insufficient diagnosis and high early mortality – both indicators of inadequate access to and delivery of care.

Cultural accessibility and acceptability are important dimensions of effective access to care. Care delivery must be aligned with the socio-cultural realities of patients and families because social representations shape individuals’ actions in the daily management of the disease.^6^ Improving T1D outcomes therefore requires attention to social representations at the community and family level.

Political and socioeconomic factors, including social representations in policy and care delivery, also influence the development and enforcement of policy and regulations and the practice of healthcare professionals. These policy- and practice-level effects shape care delivery – including interactions between healthcare professionals, children, and parents – and the impacts of T1D care delivery on patient and families’ outcomes.^6,7,8,9,10^ In sub-Saharan Africa and Cameroon in particular, there are to our knowledge no previous studies examining how care delivery for patients with T1D is represented and put into practice at these three societal levels: political decision-makers, healthcare providers and patients within families. However, few studies have concomitantly analysed T1D and the social representations of patients, families, health professionals and policy makers, especially in sub-Saharan Africa. Existing studies generally focused on one or another specific dimension: the epidemiological prevalence and situations of ocular complications^7,11,12,13^; the political dimension concerning the priority given to human immunodeficiency virus (HIV) and acquired immunodeficiency syndrome (AIDS) to the detriment of other chronic pathologies as documented in the health sector strategy plan 2016–2027; the logic underlying the non-adherence of young patients with diabetes to dietary recommendations and structuring their eating behaviour’s.^8^

The aim in this qualitative study was to explore the management of T1D and how the role of the patient in T1D care is reflected in political and health strategies, care delivery practices, and representations of patients and their families. These three levels of social representations interact to shape access to and the nature of healthcare, the lived experience and explanatory narratives of patients and families, and the outcomes and impacts of T1D on patients, families and the health system. Together, these allow a focus on the individual patient in their family and community context and support the three groups of actors – health professionals, political leaders and families – to advance optimal health and healing for the well-being of all.

Exploring social representations of T1D among children and their families constitutes a step towards patient-centred intervention programmes and a resource for political leaders and health professionals to reconceptualise T1D care and management strategies in Cameroon and other resource-constrained contexts.

## Research methods and design

### Study design

This qualitative study included document review, in-depth semi-structured interviews, a questionnaire with open-ended questions and structured observations. There were three sites, all in the capital city, Yaoundé: the Central Hospital of Yaoundé (HCY), the Association of Young Diabetics of Cameroon (AJDC) and the Ministry of Public Health of Cameroon (MINSANTE). We followed the Consolidated Criteria for Reporting Qualitative Research (COREQ)^14^ (Appendix 1). Data triangulation^15,16^ allowed us to obtain different but complementary data. We examined social representations of T1D and the role of the patient in national and international health policies, which set a framework for the implementation of care delivery; among health professionals in their daily work, and among patients and their families, regarding how they perceive T1D, care delivery, and their place in care delivery.

### Document review, study participants and data collection

We reviewed literature including Cameroonian policy documents from the Ministry of Public Health (MINSANTE), grey literature including that from WHO and diabetes organisations and published literature on the political context of T1D advocacy in Cameroon. We then developed interview and observation guides (Appendix 2). Interviews with policy makers and healthcare professionals focused on: (1) motivation and use of care delivery centres, (2) assessment of the conditions under which care delivery is carried out, (3) difficulties in implementing strategies and (4) recommendations to improve the care delivery of T1D.

Interviews with parents focused on: (1) knowledge about health centres, (2) satisfaction with care, (3) biomedical knowledge about T1D, (4) social representations and (5) recommendations. The patient questionnaire (see Appendix 3) focused on: (1) perceived usefulness of T1D care centres, (2) type 1 diabetes treatment plan, (3) knowledge about T1D and its complications and (4) social understanding of the disease.

Observations focused on interactions between patients and healthcare providers, education sessions and circulation in the centre and included taking notes during observation sessions. All data were then analysed to examine social representations of T1D by the three levels of actors.

A total of 82 people participated in the study conducted from January 2022 to April 2022: five policy makers, seven health professionals and 20 families (mother and/or father) participated in semi-structured in-depth interviews, and 50 children with T1D aged 9–18 years answered questionnaires with open ended questions (Table 1). Participants were from various ethnic groups and religious denominations, all from the urban area of Yaoundé. Participants were purposefully recruited with a 100% response rate achieved. National policy makers familiar with the policy and programmatic processes related to T1D in Cameroon were selected from different T1D-related programmes. Healthcare professionals were recruited from three public health facilities and two non-profit organisations. Children with T1D were recruited at health facilities, with the help of staff. An information sheet and consent form were sent home with each child. If parents consented to participate, the form was returned to the team at the child’s next visit. We then visited the household in person and repeated the informed assent and consent process for the child and parent, respectively. Recruitment of study participants continued until saturation was reached.^17^ Interviews were conducted in French by the first author and digitally recorded. Interviews lasted between 30 min and 60 min. Interviews were carried out in offices or in conference or meeting rooms where only the participant and interviewer were present. With parents’ consent, children were interviewed without parental presence.

**TABLE 1:** Socio-demographic characteristics of participants.

Levels	Sex	*n*	Age range (years)	Socio-Professional Category	Total
**Policy makers**	-	-	30–55	-	5
	Men	3	-	Policy makers (03); Presidents of associations (02)	-
	Women	2	-		-
**Healthcare professionals**	-	-	30–50	-	7
	Men	2	-	Doctors (03); peer educator (01); Nurse (03);	-
	Women	5	-		-
**Patients and families**					
**Children with diabetes**	-	-	9–18	-	50
	Boys	19	-	Elementary school students (37); secondary school students (10); small trades (03)	-
	Girls	31	-		-
**Parents**			30–60	Officer (02); housewives (07); teacher (01); student (01); seamstress (03); pastor (01); trade (02); civil servant (02); nurse (01)	20
	Men	3	-		-
	Women	17	-		-

Note: Numbers on brackets are the number of participants.

Daily non-participant observations over 15 days by the principal researcher of care interactions in clinical settings, as well as field notes taken during home visits and after interviews, supplemented these interviews.

### Analysis

The interviews were transcribed into MS Word software and coded through QDA Miner 5.0.31 (Provalis) following an inductive and deductive approach to thematic content analysis.^18,19,20^ Coding was done by the principal researcher after listening to all recordings, reading all transcripts at least twice, and writing analytical memos. The codes were then mapped onto the transcripts. The analysis itself highlighted convergent and divergent ideas as well as unexpected details which proved crucial for understanding the situation studied. To ensure confidentiality, all quotes from study respondents were anonymised and are referenced in this article only by professional title and social role following approval obtained from participants during a data-restitution meeting.

### Ethical considerations

This research has obtained the approval of the Regional Ethics Committee for Human Health Research of the Center (CRERSHC/C- CE Number 2172) in Cameroon and the Ethics Committee for Research in Science and Health (CERSES-21-082-P) of the University of Montréal in Canada.

All participants were informed of the purpose of the study, what it meant to participate in a semi-structured interview or answer a questionnaire, the anonymity of their response, and that they could discontinue their participation in the study at any time. Written or verbal consent was obtained at the start of each interview. For children with T1D under the age of 18, the parent’s consent was required first, then the child’s assent was sought before proceeding with the questions. Confidentiality and anonymity of patient information were respected through non-disclosure to third parties. The data were stored in a secure database.

## Results

The findings are structured at three levels: policy makers, healthcare professionals and children with T1D and families. These were reported under four major categories: (1) national policies, local strategies and international dynamics; (2) engaging patients and families in care delivery; (3) the role of the patient and relationship between patients and healthcare providers; and (4) representations of diabetes and explanatory frameworks.

### Sociodemographic characteristics of participants

Table 1 describes the 82 participants, of whom 67% were women.

#### National policies, local strategies and international dynamics

**National policies:** Cameroon’s health sector strategy for 2016–2027 focused on ‘Disease Control’ programmes in four areas: (1) major endemics (malaria, leprosy, onchocerciasis, river blindness, human African trypanosomiasis, Guinea worm, schistosomiasis); (2) chronic diseases constituting a public health problem, including arterial hypertension, type 2 diabetes, epilepsy, sickle cell disease, cancer, asthma, rheumatic diseases, deafness; (3) epidemics, in particular cholera, measles, cerebrospinal meningitis, and care delivery of emergencies because of disasters and accidents; and (4) tuberculosis, sexually transmitted infections (STI) and HIV and AIDS for which a specific programme was adopted in September 2000. A structured political commitment through a public–private partnership for T1D was initiated only in 2009 with the implementation of the Changing Diabetes in Children (CDiC) project. The 5-year CDiC project funded by the Novo Nordisk laboratory supported children with T1D in low-income countries with insulin and diabetes supplies:

‘The CDiC has increased the diagnostic capacity of the health system and has made it possible to constitute a large cohort of children and adolescents living with T1D in Cameroon and to reduce mortality linked to childhood diabetes.’ (I1, Policy maker, Age 38)

Another official in the same department reported:

‘In fact, the CDiC project consists of several clinics throughout Cameroon … it is essentially about the management of T1D in children [*at these clinics*], naturally from the moment they are screened until the age of 21. And the care is free.’ (I2, Policy maker, Age 38)

**Local strategies:** At the national level, measures are put in place to support the Cameroonian health system. Since 2009, the country has set up nine referral clinics and additional clinics to help manage routine care of children with T1D:

‘There is a clinic here at the Central Hospital, and the other clinic which is housed at the Yaoundé General Hospital. It is a set of about ten clinics: there are two in Yaoundé, there are two in Douala, there is one in the North-West precisely in Bamenda, there is one in Bafoussam, there is one in Garoua, there is one in Ngaoundéré, there is one in Maroua, there is one in Ebolowa, there is one in Limbe, yes, I believe.’ (I3, Policy maker, Age 45)

About 50 district hospitals house paediatric diabetes clinics and carry out educational activities for the prevention of hypertension, diabetes and other cardiovascular diseases. However, the MINSANTE official noted that in practice, the nine or 10 specialised clinics are not all fully functional, and more serious cases are instead referred to Yaoundé Central Hospital.

Regarding policy attention to the role of the patient in diabetes care delivery, the initial policy drafts recognise the role played by the first association of patients with T1D with the creation of an association of ‘Young Diabetics of Cameroon’ in 2013. ‘This is very important because the associations are the voice of the people. We are the voice of the government’ (I3, Policy maker, Age 45). However, the patient’s role in care delivery of T1D still seems unclear. In the 2016–2027 sectoral health strategy plan, patients are only mentioned in the context of their diabetes associations’ information and communication activities. In the draft care delivery guidelines that accompanied this plan, the patient’s contribution to care delivery was not mentioned at any level, confining patients to the status of beneficiary and receiver of information. This representation of the place of the patient does not seem to have changed since the launch of the 2016–2027 health strategy. ‘We give glucometers to these children, we give them test strips, we give them insulin, syringes and all the inputs that go with it and there is follow-up’ (I3, Policy maker, Age 45).

**International dynamics:** The Cameroonian authorities’ response to T1D has generally reflected strategies at the international level and promoted by development cooperation agencies in Cameroon. Non-communicable diseases emerged as international health priorities only in the early 2000s. Until 2012, few partners supported the Ministry of Health in this area, including the WHO, which provides important technical support in the development of strategies and the training of service providers. A political decision maker described the international contribution but was not familiar with the details:

‘The CDiC project is mainly funded … It is not the only one, but it is financed mainly by Novo Nordisk which provides us with insulin at a ridiculous cost. We are affiliated with other partners like the International Diabetes Federation [*IDF*]. Life for a Child IDF(LFAC). But I can’t tell you more.’ (I5, Policy maker, Age 52).

This international support has also influenced the mechanisms available to Cameroonian authorities, such as systems to support and supervise patient care and autonomy. The few experiences in the field of diabetes management education are also supported by the international community. This occurs through the training of health care providers by the IDF or the financing by organisations of peer education programmes at the associative level in Yaoundé. Despite these efforts, ‘There is a type 1 and 2 diabetes, hypertension, and chronic viral diseases program which was to be created but which is not yet’ (I4, Policy maker, Age 50). He continues:

‘There are several difficulties. The first is still action planning. These actions are not coordinated. We don’t know who does what. Who does how? … It is sad to say it, but Cameroon does not have a national protocol for the treatment of T1D …, there is none. If we manage to have a national protocol, we will know how the patient is taken care of throughout the territory. If we manage to have this idea of how it is taken care of and that the data goes back, we will be able to better orient the policies.’ (I4, Policy maker, Age 50)

The political, medical and social history of specific diseases has led to reflection on the participation of the patient in their care delivery. The documents and literature analysed in this study, including the 2016–2027 health sector strategy plan, reveal that in Cameroon the urgency of action against diseases such as malaria, tuberculosis or HIV and AIDS has fuelled awareness among all stakeholders – decision makers, providers and patients – of the importance of the active commitment of patients. These programmes have allowed patients to learn about the causes, course and consequences of their disease, and programmes have enabled patients to develop strategies to take charge of themselves, including being consulted in matters of health. This awareness has been accompanied and fostered by international mobilisation on the part of donors and civil society organisations, which support the creation of conditions favourable to putting patient engagement into practice. Type 1 diabetes, in contrast, is absent from the list of conditions where patient engagement is considered important.

#### Engaging patients and families in care delivery

**Patient and family engagement:** The active participation of patients in care is shaped by the connections among elements that the affected patients conceive as essential for successful care delivery. The doctor or nurse represents one of these essential elements, even if the patient knows that they must deal with the insufficient numbers and availability of nursing staff characterising the health system in Yaoundé. Most children and their families considered that being followed by the same doctor or nurse is central to optimal care delivery of their illness. Forty of the 50 children answered affirmatively to the question ‘Would you like to see the same doctor or nurse for a follow-up to today’s consultation?’ Participants’ responses, however, indicate that in practice professionals are interchangeable with no continuity of individual patient–clinician relationships. In addition, the quality of the relationship with this practitioner is a major factor shaping their experience of T1D. According to children with T1D and their families, a doctor or nurse is the one who knows social problems, their experience of living with T1D, and who masters the history of their illness since its diagnosis. ‘Since it was discovered that I have diabetes, I have been with Doctor. … She knows everything about me, I prefer to stay with her for confidence and peace of mind’ (C5, Girl, Age 16). One parent recounted how many times he preferred to wait for the arrival of his daughter’s doctor until the evening rather than being seen by another. Another child summarises a recurring theme:

‘It is he who has followed me since my birth and therefore, with the evolution of the disease, it is he who really knows if it evolves, or if it does not evolve, or if it is necessary to change treatment. He knows me well, the problems of the house where I live too.’ (C15, Girl, Age 12)

The possibility of benefiting from a personalised relationship with the same doctor or nurse is perceived as a guarantee of serenity and effective follow-up and treatment.

**Relationships with other patients:** The doctor or nurse is not the only figure with whom patients and families must negotiate a relationship to be ‘good patients’. They perceive the relationship with other patients, cared for in the same facility, as essential. Families and patients consider the knowledge held by other patients and families as complementary to that of the doctor or nurse. They also perceive other patients and families as people who can support them in managing their medical prescriptions or who can inform them about their treatments. Exchanges with peers are mainly organised in an informal setting because opportunities for group education sessions are rare:

‘I met a lady after leaving the hospital with her son suffering from T1D. We exchanged contacts and since then we share our various experiences on the disease of our children and the various remedies used.’ (P6, Woman, Age 35).

‘I went to this association of diabetics because I was told that there were other children of my age suffering from this disease … For me, it is an opportunity to exchange with other children who share the same reality as me to help me overcome my illness.’ (C3, Boy, Age 15)

Parents and patients feel invested in raising awareness and comforting other patients, a constituent aspect of what it means to them to be a patient.

#### The role of the patient: Relationship between patients and healthcare providers

Observation of interactions between patients and clinicians showed diverse relationships and practices. When compared to usual care situations in Cameroon, health care providers taking care of patients with T1D should have relatively favourable working conditions: a senior clinician reported that according to the guidelines, computers are supposed to be available, allowing for electronic medical record keeping; management protocols and local guidelines are supposed to be in place to support day-to-day work; there are individual office spaces; educational tools are available to explain the disease and its evolution; insulin and glucose monitoring strips are distributed free of charge. Such work environments might allow staff to think about the relationship with the patient in the exchange – one of mutual respect and commitment and which integrates regularity of visits and establishes the status of an active and respected patient in care, despite challenges:

‘There are certain problems: either the dad is not there, or the mom is busy with other occupations. She only knows that her child must come on Wednesday to take the medicine and does not make sure whether the child has taken his insulin or not … A good patient should be the one who follows his treatments well but who also intervenes, who can say: “Doctor …, it’s been such a long time I haven’t taken my blood sugar, or I have no more strips …”. So, he shows interest, and we also react.’ (I4, Healthcare professionals, Age 40).

However, structured observation of provider–patient interactions in the treatment setting as well as interviews with health professionals, families and patients, revealed that in reality T1D receives little support. A doctor with the CDiC project shared that insulin is available only if the clinic is enrolled in the CDiC programme. Screening, visits to health facilities, and prescribed treatments are paid for out-of-pocket, sometimes unavailable, or unreliable (e.g. for laboratory tests) when patients are not in urban areas:

‘We doctors do not have all the means we need for the care of these children; … certain examinations cannot be carried out, we refer patients to centers where the examinations are chargeable.’ (I2, Healthcare professionals, Age 42)

Caregivers are not spared from this reality:

‘Sometimes I pay for transportation there and back. Sometimes I must send money if, for example, there is a comorbidity for the care of this patient. Sometimes I must motivate the staff. It’s a lot of resources so I … When there are no strips there too, I am a little… how can I say … annoyed because I don’t know if the patient is balanced or not. For new patients who arrive when there are no strips, I have to buy to have at least their first blood sugar and see how to adjust their treatment … but it is not enough.’ (I2, Healthcare professionals, Age 42).

These conditions for healthcare work are widespread in resource-constrained public structures. Patients are seen in several facilities, not always by the same doctor or nurse. Consultation times remain short with few exchanges. The observed consultations lasted approximately 5 min – 8 min per patient. About 30 patients are received by the doctor or the nurse for half a day for the times reserved for the consultations. Information is delivered in the mode of directives: ‘Continue the treatment, monitor your blood sugar and practice sport’. Field observations also revealed that awareness-raising activities concerning nutrition and diet are delegated to a nurse whose work schedule is divided between several structures. These work situations are not conducive to disseminating knowledge about T1D and fostering effective patient participation. In practice, care relationships boil down to exchanges on isolated and unrelated points: faced with a specific problem, the nurse responds with a drug, sometimes an examination, without linking this interaction with the necessary ongoing diabetes management activities.

Clinical care is shaped and delivered based on the health policies and structures described earlier, including the very limited perspectives on the role of the patient expressed in policy documents and in interviews. In the health structures visited, the discourse of health professionals towards patients is largely one-way and focused on offering information on complications and on giving advice. Nevertheless, some health professionals surveyed mentioned other aspects such as the acceptance of the disease, the financial, nutritional, cultural problem and the patient’s entourage which are obstacles for the care of the patient and shape patients’ and families’ decisions. Psychosocial aspects of T1D care (social support, diabetes distress, stigma, mental health) are sometimes mentioned during the encounters to maintain the patient’s confidence.

Faced with these constraints and realities outside of immediate biomedical actions, families and patients turn to social practices based on their knowledge of the disease. Decisions and behaviours are motivated by interactions with the doctor, but medical advice is not the only factor informing the family’s decisions to access health care or not:

‘At the hospital the doctor gives recommendations, it’s true, but it’s me who lives with my son. It’s not all that the doctor will say that I would do since medicine does not cure everything.’ (P13, Woman, Age 45).

An adolescent living with T1D explained:

‘The doctor gives me advice that is good for my health, but in my family, there was this kind of disease, and it was treated without resorting to modern medicine.’ (C10, Girl, Age 16)

These illustrations demonstrate that the actors and their entourage develop different forms of social representations and find an explanatory framework justifying their decisions to access health care or not.

#### Patients’ and families’ representations and explanatory frameworks for diabetes

**Traditional representations of type 1 diabetes:** The traditional or lay representations of T1D incorporated but transformed biomedical notions related to the biochemistry of T1D (sugar disease), the most common identifying symptom (urine disease), and the invisibility of the causal agent (disease of the body).

***Sugar disease:*** Type 1 diabetes is perceived as the disease of dangerous sugar levels. Many children with T1D and their families interviewed recognise that certain staple foods contain sugar and attribute the origin of T1D solely to the consumption of sugary drinks, sweets and white sugar itself – hence the literal translation as sugar disease by some children with T1D and families.

***Urine disease:*** Some children with T1D and families we met literally referred to T1D as ‘urinary disease’. This name references the most characteristic symptom, and the one most frequently mentioned by children with T1D and families:

‘My son Divine here, he had diabetes when he was 4 years old. … when he came home from school he started peeing too much, so I took him to his pediatrician, the pediatrician consulted him he took his blood sugar, he said: “ooh! the child has diabetes.”’ (P10, Woman, Age 48)

This representation of an illness which is defined by the frequency of urination means that for some children and families living with T1D, a patient on insulin may believe he or she is cured when this symptom fades and may therefore stop ongoing adherence to the treatment.

***Disease inside the body***: For some children with T1D and their families, T1D is defined as the disease of the body. This name underlines the invisible nature of the disease. It cannot be seen with the naked eye; it is imperceptible. Based on this name, we also note its wide-ranging and dramatic dimension:

‘One day I noticed that my son was losing a lot of weight, he was peeing a lot, he was eating and was not gaining weight, I said that it was not normal. I took him to the hospital up here to give me medicine so he wouldn’t get up too much at night to pee because I had never seen anyone with this kind of disease.’ (P16, Man, Age 56).

Many of the children’s narratives suggest that their classmates have set beliefs as well as negative representations. Some equate it with a disease of the elderly, while others perceive it as a contagious disease or a mystical disease. ‘Most of the time, everyone thinks it’s a mystical disease. Many did not know that children my age could have diabetes’ (C12, Girl, Age 12).

**Beyond a disease: Situating T1D in life context:** Parents’ representations express practical and moral concerns. First, parents stress the difficulty of managing the constraints of diabetes and the fears related to diabetes in terms of risk. Some parents said:

‘We don’t always have the means and we don’t know all the prohibitions … we don’t know what to do in times of crisis, panic, we fight. At this level we fight, it’s not easy for us her parents but it is necessary to avoid the worst.’ (P1, Man, Age 50)

‘Since he was ill there have been financial problems. I am a housewife; how much is the housewife’s salary? Once he complained to his doctor: I want to eat this, they tell me not to eat. I must fight to make his meals different from the others so that he has something to eat if I don’t want to see him suffer. But it’s hard as I tell you.’ (P11, Woman, Age 49)

Anxiety is also expressed by parents. Their child’s diabetes makes them feel responsible and guilty:

‘The child you see here was only diagnosed with diabetes when he was 9 years old. I didn’t know that a child could have diabetes and at the hospital they said it was hereditary. So, I said to myself that it may be in my family or that of his father that he had this disease …’ (P14, Woman, Age 38)

Other parents mentioned the frustration and depression they had to overcome upon learning of their child’s illness. One parent shared this in these terms:

‘I did not know what diabetes is. I once noticed that my son was losing a lot of weight, he was peeing a lot, he was eating and not gaining weight, I said that was not normal. I took him to the hospital because I had never seen anyone with this kind of disease … At the beginning it was difficult … it was catastrophic. That’s what made me lose weight until today because I didn’t accept that. It was terrible! My child saw ghosts, the devil everywhere, went … I can’t tell you.’ (P15, Woman, Age 42)

Some parents expressed sadness and keen awareness of their limitations in the face of their child’s state of health:

‘I feel alone with him, and I don’t have stable work, so I struggle day to day. That’s why I’m going to tell you again, it’s in the hands of God, the care of this child. Because if I had to buy the drugs, maybe I couldn’t.’ (P8, Woman, Age 35)

Not all actors face the same financial problems. Some of the respondents benefit from financial aid, while others encounter enormous difficulties in supporting themselves, as was the case for this mother.

Finally, parents raised the involvement in the therapeutic education of their children as a constraint. Most of the parents interviewed stated that they do not often have time to attend therapeutic education sessions with their children, sometimes phrasing the time constraint as ‘not yet’.

Children’s representations are more concrete and immediate. They express their experience and tell the story of their diabetes with what to do and how they live daily. For example, more than half of the patients initially thought that an unhealthy diet that was too sweet or too rich in fat was the cause of their diabetes.

‘When I arrived at the central hospital, well … the doctors advised me that the problem is not the salt or the cube that we put on the food. This is where I started eating like everyone else at home. But what I can’t take is sugar … that’s how it is.’ (C22, Girl, 12 Age)

Type 1 diabetes is perceived by some of them as a complicated disease, an embarrassing disease that limits their lives and freedom, for example, through the frustrations caused by their required eating habits. ‘For me, diabetes is a limit to my lifestyle. There are things other people do that I can’t do, like food’ (C25, Girl, Age 13).

Some children with T1D expressed concern about death, especially when a family member who had diabetes has died:

‘I was overwhelmed by the idea of now being considered diabetic when I was diagnosed and having to go through what my mother went through to finally die of complications.’ (C39, Boy, Age 17)

Another child recounted:

‘There is a child who died last year in my neighbourhood because of diabetes. He ate too much sugar and then he had diabetes. Then they cut off his leg.’ (C19, Girl, Age 14)

Financial constraints within the family are recognised by children as an obstacle, particularly among young people from large families:

‘In some cases, I eat what we prepare at home. Because what I eat is a little more expensive.’ (C27, Girl, Age 14)

‘The remedy sometimes ends up in the hospital and you must buy it. It’s expensive and I must work until late to have money since his father is no longer living and I have no other support.’ (P8, Woman, Age 35)

## Discussion

In this study, children with T1D and their families continue to face limitations in terms of care despite the existence of numerous laws and policies adopted to help patients and families in Cameroon. Multiple forms of cultural barriers and policy implementation challenges still exist, preventing patients and families from being fully active participants in care. The participants described the resources available, and the local strategies put in place in health structures and within families. Understanding the patient’s role requires the patient’s commitment and his relationship with others. Children with T1D and their families expressed their perception of living with the disease.

In sharing their representations and explanatory frameworks of T1D, patients and families suggest that T1D obeys what Doukouré refers to as the principle of double causality^21^ (natural and supernatural causes) and social determinants of health.^22^ Baudrant et al.^23^ explore the concept of representation in developmental psychology and social psychology. In cognitive psychology, the term designates the personal interpretation of the phenomenon. In social psychology, it designates a collective interpretation of a phenomenon. Moscovici^24^ defines social representation as ‘a particular mode of knowledge whose function is to develop behaviour and communicate between individuals’. These representations are to some extent external to individuals. They shape attitudes and behaviours. Herzlich^25^ proposes representations as phenomena of a dual nature. The statements collected on health may reflect individual opinions or the way society has constructed these ideas. Social representations are built at all levels of society – they are not limited to ‘community’ or ‘lay’ representations. The social representations articulated in policy and manifest in care delivery also play critical roles in shaping practice and outcomes.

Through the analysis of MINSANTE policy documents and literature, we were able to analyse the voices of different groups of policy makers with respect to relationships between the role of the patient in care delivery, global health politics and priorities, and the state’s implementation of strategies for people with diabetes. Type 1 diabetes has long been neglected in national and international health priorities. Slow political response, low material, human and financial resources, and the stigmatisation of children with T1D have limited both the quality of T1D care and the opportunities for the development of an active patient. However, decision makers are aware of these challenges.

We considered the care context, including healthcare professionals’ interactions with patients and families. Health professionals are figures on which patients and families rely to engage and become aware of their state of illness. Healthcare professionals expressed that insufficient material and financial resources constitute both obstacles to adequate medical care and to supporting patients and their families to be active partners in disease management. These barriers to effective clinical and cultural access make it almost impossible for clinicians to offer comprehensive and caring healthcare, even when clinicians are well aware of the social, economic and emotional burdens of T1D.

When a patient and their family visit a health centre, they interact with the healthcare provider and the health system based on their own social representations of the disease. Patients’ and families’ representations of the causes and implications of T1D and their actual practices of engagement in care are also shaped by the practices of health professionals and the way in which patients would ideally wish to engage in care. Aware both of the role that patients must play and of the weaknesses of the health system, these patients are far from remaining passive and seek to manage their pathology with the means they are given and with the means they build based on socially constructed representations, for example, by making rearrangements in their daily life.^26^ Other resources such as the relationship with other patients are highlighted. Depending on the nature of their pathology, patients and families do not have the same management mechanisms or resources to manage their health problems. Some negative or erroneous representations of T1D combined with the financial and social constraints of treatment and diet lead to stigmatisation and exclusion for some to young people with T1D. However, even though patients and families do not receive sufficient institutional and clinical support, their expectations and their practices in the personal management of T1D echo the models of care recommended for chronic disease.

In sub-Saharan Africa, as in Cameroon, given the low rate of education and the lack of knowledge of chronic diseases, knowledge of T1D is low: patients do not have a broad knowledge of the pathology from which they suffer. In addition to mass campaigns, interviews or brief motivational counselling^27,28^ with patients could enable them to learn more about the disease and its associated risks. Type 1 diabetes is still perceived by patients and their families as a disease of diverse realities, some of which are in conflict with biomedical understanding. A recent study of the perceptions and representations of diabetes in a community in Cameroon reported that community members represent diabetes by referring to the cultural models of the society to which they belong.^6^

### Strengths and limitations

This study takes an in-depth look at the challenges and concerns of these three groups and provides new insights based on triangulating rich data within and across societal levels. This Cameroonian study contributes to better understanding and operationalising the call for a paradigm shift in a specific context, as the general principles of patient-centred care of chronic NCDs must be implemented in specific socio-cultural contexts. The patient and family narratives of T1D in its broader emotional, moral and social contexts poignantly reveal how negatively T1D is experienced in Cameroon, even by families and patients actively receiving specialised care. They also reveal patients and families actively seeking to be engaged in – indeed, at the centre of – care.

The study had several limitations affecting its generalisability. The sample included only select children with T1D enrolled in the data collection sites selected for this study, all of which were within the national capital area. The issues presented are limited to the context of the capital of Yaoundé. They remain to be studied outside the capital and its specialised structures, in Cameroon as in other low-income countries, and among children and families who have not yet been enrolled in care for T1D. We did not collect the detailed demographic information that would allow a deeper, intersectional equity analysis. The sample of healthcare professionals and decision makers was limited to those already active in T1D policy and care delivery; it is likely that those outside of this small group face even greater challenges in offering comprehensive, respectful, patient-centred care with the active engagement of patients and families.

## Conclusion

This study builds on and extends studies carried out in Cameroon and elsewhere. Unlike studies that address one dimension of T1D, we have put into context medical, social and political dimensions including the image that young patients and their families have of their disease, as well as the perceptions of political leaders and health professionals.

The contributions of both political leaders and health professionals are necessary to implement interventions, from education of patients and families about T1D to improving access to and utilisation of care through addressing longstanding barriers to access. This can be done through promoting dialogue and collaboration between health care professionals from various disciplines, political leaders and families. Specific actions include improving the availability (and quality) of testing, care and insulin; ensuring adequate numbers of well-trained and supported health workers and acting across sectors to address critical social determinants such as food and transport. Such interventions may allow children and families to modify their system of representations by enhancing their understanding of the biomedical aspects of T1D and its care. In addition, policy documents that shape both practice and social representations could go beyond representing patients as passive objects of instruction and treatment and recognise and support patients and families as active players at the centre of care. Finally, actively listening to the voices of children and families can help all actors – from children to political decision makers – keep the human costs of T1D at the forefront of efforts to ensure that T1D is a manageable and not devastating condition. Our findings may support political leaders and health professionals, working closely with children and families, to better identify relevant, socially and culturally resonant content for T1D programmes.

## Appendix 1: Consolidated criteria for Reporting Qualitative research (COREQ) checklist

**TABLE 1-A1:** Evaluation of qualitative research according to COREQ’s 32 criteria.

**Domain 1: Research team and reflexivity**	
*Personal characteristics*	
1. Interviewer or facilitator	Yes
2. Credentials (of researchers)	Yes
3. Occupation (of researchers at the time of the study)	Yes
4. Gender	Yes
5. Experience and training	Yes
*Relationship with participants*	
6. Relationship established	Yes
7. Participant knowledge of the interviewer	Yes
8. Interviewer characteristics	Yes
**Domain 2: Study design**	
*Theoretical framework*	
9. Methodological orientation and theory	Yes
*Participant selection*	
10. Sampling	Snowball
11. Method of approach	In person, attending physician, parents
12. Sample size	83
13. Non-participation (from selected sample)	None
*Setting*	
14. Setting of data collection	hospital, care centre and association, home, office
15. Presence of non-participants	No
16. Description of sample	Yes
*Data collection*	
17. Interview guide	Yes
18. Repeat interviews	Yes
19. Audio or visual recording	Yes
20. Field notes	Yes
21. Duration	30–60
22. Data saturation	Not applicable
23. Transcripts returned	No
**Domain 3: Analysis and findings**	
*Data analysis*	
24. Number of data coders	1
25. Description of the coding tree	No but available
26. Derivation of themes	Predetermined
27. Software used	Yes
28. Participant checking	Yes
*Reporting*	
29. Quotations presented	Yes
30. Data and findings consistent	Yes
31. Clarity of major themes	Yes
32. Clarity of minor themes	Yes

Developed from: Tong A, Sainsbury P, Craig J. Consolidated criteria for reporting qualitative research (COREQ): A 32-item checklist for interviews and focus groups. Int J Qual Health Care. 2007;19(6):349–357. https://doi.org/10.1093/intqhc/mzm042

## Appendix 2: Guidelines for in-depth semi-structured interviews and non-participant structured observations

**TABLE 1-A2:** Semi-structured interview guide with policy makers and healthcare professionals.

Date:	Location:
Interviewer(s):	
Interviewee’s title and organisation:	
Sex of interviewee:	Status:
F/M	
Age:	Level:

### Introduction

Thank you for agreeing to be interviewed today. This interview will take approximately 1 h of your time. The information that you will provide will be kept confidential. Your responses will be anonymised unless you want to be identified. Your responses will only be shared with other research team members and will be compiled with other answers from various participants.

I am going to ask you questions about your involvement in the development of policies and the implementation of strategies in health services and local associations concerning the care delivery of type 1 diabetes (T1D) in children and families. At any time, you can ask for clarification if the questions seem unclear to you.

### Anonymity

Despite being recorded, I would like to assure you that the discussion will be anonymous. The tapes will be kept safely in a locked facility until they are transcribed word for word, then they will be destroyed. The transcribed notes of the interview will contain no information that would allow individual subjects to be linked to specific statements. You should try to answer and comment as accurately and truthfully as possible. If there are any questions or discussions that you do not wish to answer or participate in, you do not have to do so; however, please try to answer and be as involved as possible.

### Guiding questions

- Can you explain to us to what extent your professional status gives you satisfaction? (*Explore the organisational challenges, on the roles, the respect of the schedule of the activities as planned*).
- How do you assess your working conditions in the context of activities related to the care delivery of T1D? (*Explore remuneration, travel, reception of families, training provided and received, the sufficiency of material and human resources*).
- What can you tell us about the interest of families in care centres?
- What can you tell us about the role that your structure plays in the country’s health care policy in relation to T1D in Cameroon?
- Interactions with the other actors involved: MINSATE, international organisations, hospital institutions, local and profit-making associations (*Explore whether they operate in synergy*).
- Can you tell us about the difficulties you encounter when carrying out the activities and the measures taken to anticipate the traditionally known obstacles? (Explore material and human resources, communication/information, adaptation of places, distance).
- In your opinion, what are the reasons why the families of children with T1D come back during the sessions, for example, the therapeutic sessions or the information sessions organised by your structure in connection with T1D? Justify your answer.
- What is your opinion on the interaction between local associations and other government, hospital, or international programmes? (Explore the possibility of integrating these programmes into the functional structure of local associations or vice versa).
- What are the threats (contextual elements) that have influenced/could influence actions related to the management of T1D in your organisational structure? (Explore the measures taken to circumvent them).
- What are the opportunities to facilitate the integration of other activities and programmes into your organisational structure?
- What recommendations can ensure effective care within your organisational structure?

### Conclusion

- Thank you for participating. This has been a very successful discussion.
- Your opinions will be an asset to the study.
- We hope you have found the discussion interesting.
- I would like to remind you that any comments featuring in this report will be anonymous.
- If you would like to review some of the accuracy of your statements when we will write the report, please let me know if you would like to be contacted. If yes: email or local phone number.

**TABLE 2-A2:** Semi-structured interview guide with parents of children with type 1 diabetes.

Date:	Location:
Interviewer(s):	
Interviewee’s title and organisation:	
Sex of interviewee:	Status:
F/M	
Age:	Level:

### Introduction

Thank you for accepting to talk to us today. This interview will take approximately 1 h of your time. We can take breaks in between if needed, please let us know. The information that you will provide will be kept confidential. Your answers will not be associated with your name, unless you want to be identified. Your answers will only be shared with other research team members and will be compiled with other answers from various participants with disabilities.

I am going to ask you about your perceptions (i.e. ideas, points of view) on how laws, health policies are implemented in health services in relation to the management of type 1 diabetes (T1D). At any time, you can ask for clarification if the questions seem unclear to you.

### Anonymity

Despite being recorded, I would like to assure you that the discussion will be anonymous. The tapes will be kept safely in a locked facility until they are transcribed word for word, then they will be destroyed. The transcribed notes of the interview will contain no information that would allow individual subjects to be linked to specific statements. You should try to answer and comment as accurately and truthfully as possible. If there are any questions or discussions that you do not wish to answer or participate in, you do not have to do so; however please try to answer and be as involved as possible.

### Guiding questions

- How were you informed of the existence of T1D care centres in your region?
- Can you explain to us the reasons why you agreed to have your child monitored and cared for in this centre rather than another? (Explore who decides in the household).
- What can you tell us about your interest of families vis-à-vis these care centres or strategies put in place by organisations, hospital structures and the government?
- Can you tell us about the interaction between you and the other actors involved (other families, care providers, representatives of organisations and the Ministry)?
- What is your perception of the strategies put in place by organisations to manage T1D? And what image do you have of your child’s T1D?
- Can you tell us about the difficulties you encounter during the support sessions within these sites? According to you, what are the obstacles related to good care delivery of T1D in Cameroon? (Explore material and human resources, communication/information, adaptation of places, distance.)
- What steps are your family or other families around you taking to overcome these obstacles?
- Can you tell us about the difficulties you encounter in administering insulin to your children or that they encounter? (Explore the reasons for poor adherence or follow-up in care).
- In your opinion, what are the reasons that can prevent a child aged 18 and under from benefiting from free or adequate care in these various sites?
- What are your recommendations to guarantee effective care from organisations and allow families with diabetic children to have quick, if not free, access to care in these different sites?

### Follow-up during focus groups and assignment of questionnaires

Before we separate, if necessary, would you be interested in your child being part of a discussion group with other children with T1D and to answer our questions to deepen our understanding of what we have just talked about.

Y/N

### Conclusion

- Thank you for participating. This has been a very successful discussion.
- Your opinions will be a valuable asset to the study.
- We hope you have found the discussion interesting.
- I would like to remind you that any comments featuring in this report will be anonymous.
- If you would like to review some of the accuracy of your statements when we will write the report, please let me know if you would like to be contacted. If yes: email or local phone number

### Non-participant structured observation guide

Detailed field notes should be kept for each visit. This tool includes a checklist of points to consider when observing. We will write detailed descriptions of what we will see.

- During the observation, special attention should be paid to the ‘who, what, where and when’ of the different processes taking place on the site. Here, a detailed description of the stages of children with T1D and families on average between their arrival and their departure from the health centre will be made. What, with whom, where and when should be derived from observation of several diabetic families and should be summarised, for example, in tabular form
- After these more general observations, the observation should focus on the interactions between health care providers. Families of children with diabetes and others.

### Other observations

✓ About the context
✓ About the time required to perform certain processes
✓ About processes and interactions
✓ About the actors involved
✓ Reflexivity
✓ Other issues

**Table:**

Items	Observations
**Where (Location)**	
**Attention to accessibility features (physical and communication)**	
**When (Date and time)**	
**What (Activities)**	
**Who (People and social identities)**	
**How (Attitudes, facilitators, attitudes, etc.)**	

## Appendix 3

**TABLE 1-A3:** Questionnaire for children with type 1 diabetes.

**Questionnaire number: Telephone number of families and patients**		
**1- Sociodemographic data**		
1	What is your age and sex?	………years F/M
2	What is your level of education?	□ None □ Primary □ College □ Secondary □ Academic □ Other………
3	What is your marital status?	□ Married □ Divorced □ Widowed □ Single
4	What is your profession?	□ Housewife □ Civil servant □ Pupil □ Student □ Other ……………
5	Place of residence?	□ Urban □ Rural
**2- Level of knowledge of the interest of T1D treatment centres**		
1	Do you have any information on T1D treatment and its benefits?	□ Yes, specify………… □ No, why……………
2	Your source of information?	□ Health centre □ Internet □ Radio, TV □ Family □ Neighbours □ Other
3	How many care sessions do you think a child or family with a child with diabetes should attend? ……….	
4	What do you think are the danger signs for a child with T1D?	□ Fever □ HTA □ Anaemia □ Death □ I don’t know
**3- Current support process**		
1	Did you do a check-up during the period of illness?	□ Yes, specify □ No, why
2	Do you have medical coverage? □ No □ Yes, which……………….	
3	What are the days reserved for the management of T1D in health centres that you know? …………………………	
4	Are these days right for you? □ Yes □ No, why……………………	
5	How long does it take to get to your health centre?	□ < 15 min □ [15–30] □ [30 min –1 h]□ > 1 h
6	What time of day is right for you?	□ Morning □ Afternoon
7	What means of transport do you use to get to the health centre?	□ On foot □ Personal transport □ Public transport □ Other……………
8	How much do you spend per month?	□ < 1500 CFA francs □ [1500–2999 CFA francs] □ [3000–4499 CFA francs] □ > 4500 CFA francs
9	Have you ever attended awareness sessions by other care providers other than nurses during this period?	□ Yes, how many…………… □ No, why………………
10	Would you like to contact the same doctor or nurse for a follow-up to today’s consultation?	□ Yes, why…………… □ No, why………………
11	How long have you been attending support sessions at this site?	
12	Your parents or you had received an SMS or a phone call to go to a specific health centre? If yes, did the SMS help you remember to come to the health centre?	
13	What advice did the doctor or nurse give you?	
14	Did you feel that the health care providers (doctors, nurses or therapists) were interested or concerned about your condition?	
15	Are there any medications or insulin injections that the care managers should have given you for free or things to do but are out of stock or not done?	
16	When is the date of your next visit to the centre? (None; 1 day, 1 week, 1 month, 2 months, 3 months later)	
**4- Level of knowledge of type 1 diabetes and its complications**		
1	Have you ever heard of childhood diabetes?	□ Yes □ No
2	If yes, what is your source of information?	□ Health centre □ Internet □ Radio, TV □ Family □ Neighbours □ Other……
3	Have you ever faced childhood diabetes in your family before?	□ Yes □ No
4	What do you think are the dangers of childhood diabetes in a child’s life?	□ Other complications □ I don’t know
5	How do you think the diagnostic test for T1D is done?	□ Blood test □ Urine test □ Other ………. □ I don’t know
6	Do you know the symptoms of childhood diabetes?	□ Yes □ No □ I don’t know
7	In your opinion, what is the best place to monitor children with T1D?	□ At the health centre □ At the hospital □ Other …………… □ I don’t know
8	Do you think a child with T1D should follow a special diet?	□ No □ I don’t know □ Yes
9	Is physical exercise beneficial for diabetic children?	□ No □ I don’t know □ Yes
**Questionnaire number: Telephone number of families and patients**		
**5- Social representations**		
1	What image have you had of diabetes since you were diagnosed? (Different *expressions to qualify the disease*)	
2	How have the other children around you and even your parents looked at you since you were diagnosed with the disease?	
3	How do you interact with others to overcome people’s ideas about your illness?	

T1D, type 1 diabetes.
